# Synthesis and Gas-Sensing Performances of Mn_3_O_4_-Decorated Cr_2_O_3_ Nanoparticle for H_2_S Detection

**DOI:** 10.1155/tswj/8845797

**Published:** 2025-03-24

**Authors:** Hawraa Kassem Hami, Hussain Ismail Abdulah

**Affiliations:** Department of Chemistry, College of Science, Mustansiriyah University, Baghdad, Iraq

## Abstract

Mn_3_O_4_ decorated with Cr_2_O_3_ was prepared via a cost-effective and straightforward photolysis method and utilized as an H_2_S gas sensor. The nanocomposite exhibited excellent chemical sensing properties toward H_2_S gas at 200°C, achieving a sensitivity of 89.93. A proposed sensing mechanism highlights the synergistic roles of Mn_3_O_4_ and Cr_2_O_3_ in gas detection. This study underscores the potential of Mn_3_O_4_–Cr_2_O_3_ composites in industrial and environmental monitoring applications, offering a reliable and efficient solution for detecting hazardous gases.

## 1. Introduction

Air is essential for all living organisms, and without it, life on our planet would not exist. However, increasing human activity and industrial development have led to severe environmental issues, particularly air pollution [1]. Air pollution significantly impacts society's well-being and even threatens the survival of humanity. The presence of air hazards causes health damage like cardiovascular diseases, respiratory illnesses, cancer, and in some cases death. Additionally, air pollution leads to environmental damage, including acid rain, climate change, and the depletion of the ozone layer [2, 3].

One approach to addressing air pollution is by using gas sensors, devices that sense specific gaseous species or molecules by altering one or more of their physical properties, such as mass or electrical conductivity, and converting these changes into a quantifiable electrical signal. Gas sensors are used in the chemical industry, natural gas applications, and environmental monitoring [4, 5].

Nanomaterials have emerged as a promising solution in various scientific and industrial fields, due to their unique properties that differ significantly from traditional materials. These materials are characterized by their small size (typically less than 100 nm) [6], providing a high surface area relative to their volume and enhancing their chemical and physical reactivity. One of the most notable applications of nanomaterials is their use as gas sensors [7, 8].

Among nanomaterials, nanoscale metal oxide nanoparticles (MONPs) play a pivotal role as gas sensors due to their distinctive features [9]. These include high sensitivity, allowing them to detect even small amounts of gases, and their thermal and chemical stability, which makes them suitable for use in harsh environmental conditions [10, 11]. They are also selective, lightweight, cost-effective, and easily miniaturized; such properties enable their use in various applications such as air quality monitoring, toxic gas alarm systems, and environmental industries. However, MONP-based sensors have limitations, such as susceptibility to humidity and the need for high temperatures, typically ranging from 100°C to 400°C [12, 13].

Chromium oxide (Cr_2_O_3_) is one of the most widely used materials in many applications, valued for its low toxicity, heat resistance, and adaptability to environmental conditions [14, 15]. It exhibits both n- and P-type semiconductor behavior and has been employed as a sensor for detecting gases like H_2_S [12], *n*-butanol [16], NO_2_, and NH_3_ [15].

Manganese oxides (MnO_x_) are another class of materials extensively utilized in various applications. Among these, Mn_3_O_4_ (Hausmannite) is a mixed oxide containing Mn^2+^ and Mn^3+^, which makes it particularly attractive for its distinctive structure, unique electrical and magnetic properties, environmental friendliness, and high catalytic efficiency. These attributes make it suitable for numerous technological and environmental applications [17, 18] Sensors based on Mn_3_O_4_ have been used to detect gases such as H_2_S, CO, NH_3_ [19], H_2_, and CO [20].

This study involves the preparation of Mn_3_O_4_ decorated with Cr_2_O_3_ MONPs in an easy, fast, and environmentally friendly way using a UV source. The combination of Mn_3_O_4_ and Cr_2_O_3_ enhances the comprehensive efficacy of the sensor, making it the best choice for H₂S gas detection applications, as it combines high sensitivity, fast response, and operation at appropriate temperatures.

## 2. Material and Methods

### 2.1. Chemicals

Chromium nitrate nonahydrate Cr(NO_3_)_2_·9H_2_O (≥ 99.0%) was supplier by Merck, India. Manganese dichloride Mn_2_Cl_2_·4H_2_O (≥ 99.0%), potassium hydroxide (KOH) (≥ 97.0%), PVC, and N-methyl-2-pyrrolidone were supplied by Sigma-Aldrich. Deionized water (DW) was utilized to form all aqueous solutions.

### 2.2. Methods

Pure Cr_2_O_3_, Mn_3_O_4_, and Mn_3_O_4_-decorated Cr_2_O_3_ MONPs were prepared using the photolysis method [21, 22]. To synthesize, a solution of 0.1 M Cr(NO_3_)_2_·9H_2_O and 0.2 M MnCl_2_·4H_2_O was mixed and stirred for 30 min. The mixture was then placed in a photolysis cell, which consisted of a quartz tube with a UV light source (125 W, *λ* = 365 nm) inside a Pyrex tube as a reactor, and irradiated for 30 min in an ice bath. A drop of KOH (6 N) was added to form a brown precipitate. This was washed multiple times with DW, then separated using a centrifuge at 4000 rpm, dried at 80°C for several days, and then calcined at 600°C for 3 h.

### 2.3. Manufacture of Gas Sensor and Measurement

**Figure 1:**
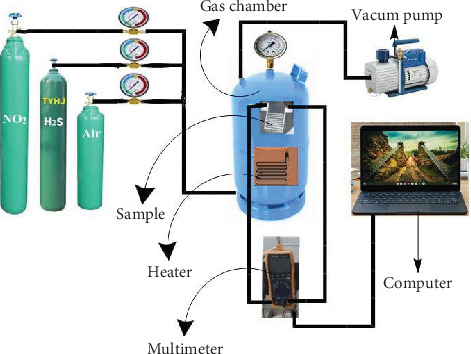
Schematic diagram of gas sensor experiment.

The gas sensor was manufactured by mixing an appropriate amount of Mn_3_O_4_-decorated Cr_2_O_3_ MONs powder with PVC as a binder, crushing it manually in a mortar, and then adding drops of the solvent (N-methyl-2-pyrrolidone (C_5_H_9_NO)) to make a paste, which was then distributed evenly on glass slides (2 × 3 cm) that have been washed with several washing solutions to remove contaminants from their surfaces. Then, the film was placed in an oven at a temperature of 100°C for 4 h to dry the film [12]. A homemade sensor device was used to determine the sensor's response at the operation temperature range of 150–250°C. H_2_S gas was employed with a mixing ratio of 10% H_2_S/air (Figure 1). All samples had a bias voltage of 6 V. The sensitivity (*S*%) was computed using the equation:

(1) $$\begin{array}{c} S\%=\frac{Rg-Ra}{Rg}*100\% \end{array}$$

where *Rg* and *Ra* represent the membrane electrical resistance in the presence of gas and air [23].

## 3. Result and Discussion

### 3.1. X-Ray Diffraction (XRD) Analysis

**Figure 2:**
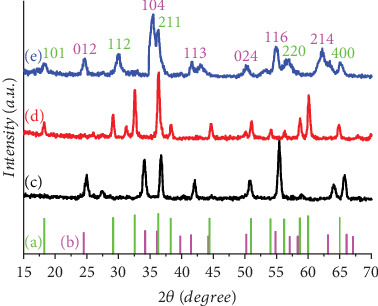
XRD patterns of (a) Mn_3_O_4_ MONPs (JCPDS No. 24-0734), (b) Cr_2_O_3_ MONPs (JCPDS No. 96-900-8085), (c) Cr_2_O_3_ MONPs, (d) Mn_3_O_4_ MONPs, and (e) Cr_2_O_3_–Mn_3_O_4_ MONPs.

XRD analysis revealed the presence of characteristic peaks indicating the formation of a mixture of crystalline phases of Cr_2_O_3_ and Mn_3_O_4_. The Cr_2_O_3_ peaks correspond to the rhombohedral phase (JCPD No. 96-900-8085) [24], where the peaks appeared at 2*θ* = (24.6, 33.8, 41.6, 50.1, 54.89, 63.2) which are compatible with (012, 104, 113, 024, 116, and 214). As for MnO, it appeared as a tetragonal Hausmannite phase (JCPDS No. 01-089-4837) [20], where the peaks at 2*θ* = (18.29, 29.2, 36.3, 44.63, and 64.8) can be compatible with (101, 112, 211, 220, and 400) (Figure 2).

Nested values reflect the presence of crystalline overlap between the phases; this is due to the nanoscale nature of the material, which exhibits intercalation between the two oxides at the interface boundaries. In addition, the width of the peaks (FWHM) indicates the small size of the crystals, which increases the interference effect and shows broader and less sharp peaks. The average particle size determined from the Debye–Scherrer formula [25] is Cr_2_O_3_ = 13.68 nm and Mn_3_O_4_ = 31.4 in the Mn_3_O_4_-decorated Cr_2_O_3_ MONPs.

### 3.2. EDX and FESEM Analysis

**Figure 3:**
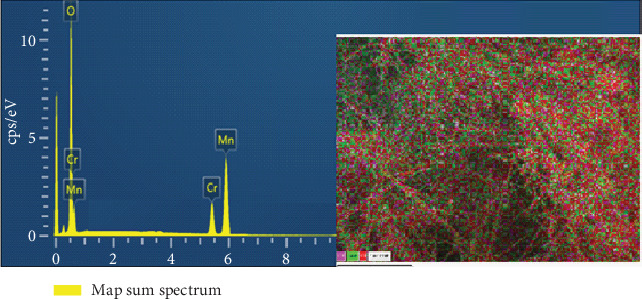
EDX spectra and EDX map of elements in the structure of Mn_3_O_4_-decorated Cr_2_O_3_ MONPs.

The EDX analysis confirmed chromium, manganese, and oxygen as the primary elements. The distribution map (Figure 3) reveals that Mn_3_O_4_ appeared in the form of nanothreads extending throughout the structure while Cr_2_O_3_was distributed on the surface of Mn_3_O_4_. This confirms that Cr_2_O_3_ covers or decorates the Mn_3_O_4_ surface, thereby enhancing the effective surface area and providing strong interactions between the two materials.

**Figure 4:**
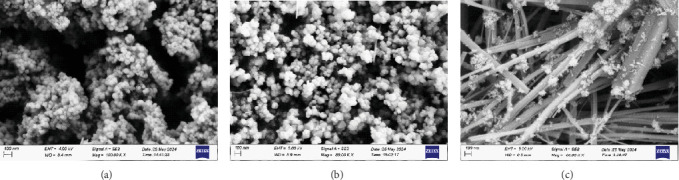
FESEM image of (a) Cr_2_O_3_ MONPs, (b) Mn_3_O_4_ MONPs, and (c) Mn_3_O_4_-decorated Cr_2_O_3_ MONPs.

The surface topography of the prepared nanomaterial as shown by FESEM images demonstrates distinct characteristics. Pure Cr_2_O_3_ MONPs (Figure 4a) exhibit a spherical shape with some voids. In contrast, the FESEM image of Mn_3_O₄ MONPs (Figure 4b) reveals a homogeneous distribution with a polyhedral shape. The presence of spaces between the particles suggests that this oxide has potential applications as a gas sensor.

The FESEM image of Mn_3_O_4_-decorated Cr_2_O_3_ MONPs (Figure 4c) shows MnO threads that are cross-linked and connected, forming a porous nanonetwork. Furthermore, Cr_2_O_3_ appears as small particles visible on the surface of these threads. This distribution indicates that Cr_2_O_3_ forms a layer covering the surface of the MnO threads [26].

The surface texture of the threads shows noticeable roughness and irregularity, suggesting a strong interaction between the components of the compound, whether mechanical or chemical. This nanostructure enhances the physical and chemical properties of the composite, making it suitable for various applications, including catalysis, energy storage, sensing, or use in magnetic materials [27].

## 4. Gas Sensor Characteristics

**Figure 5:**
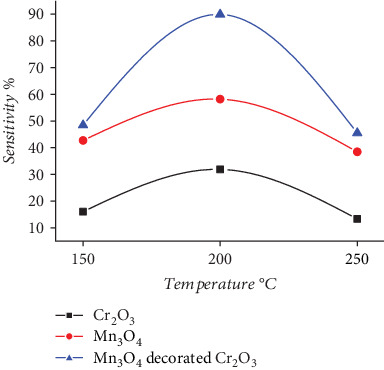
Sensitivity percentage of H_2_S gas for Cr_2_O_3_, Mn_3_O_4_, and Mn_3_O_4_-decorated Cr_2_O_3_ MONPs versus operation temperatures.

**Figure 6:**
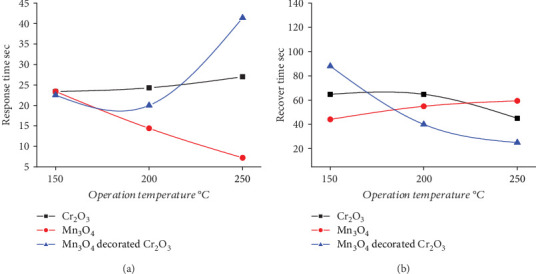
(a) Response and (b) recovery time of H_2_S gas versus operating temperature.

Semiconductor oxide–based sensors are considered among the most crucial type of gas sensors [28, 29]. To investigate the behavior of the sensors and the temperature effect on the phenomena of adsorption, reaction, and desorption, it is essential to first determine the optimum operating temperature [30, 31]. The sensitivity of Cr_2_O_3_, Mn_3_O_4_, and Mn_3_O_4_-decorated Cr_2_O_3_ MONPs was evaluated by exposing them to H₂S gas (Figure 5). The optimum sensing temperature was found to be 200°C. As observed in Table 1, Mn_3_O_4_-decorated Cr_2_O_3_ MONPs exhibited a sensitivity *S*% of 89.93, with a response time of 20 s and recovery time of 40.1 s as shown in Figure 6a,b reservedly. These results are due to the excellent catalytic properties of Mn_3_O_4_ and Cr_2_O_3_, which enhance the interaction between the gas and the sensor's surface, facilitating a fast and accurate response. The synergy between Mn_3_O_4_ and Cr_2_O_3_ improves performance by enhancing the electrical conductivity. Additionally, Cr_2_O_3_ is characterized by chemical and thermal stability, ensuring the reliability of the sensor in difficult operating conditions [14, 19].

When comparing the gas sensitivity results of H_2_S with other compounds containing Mn_3_O_4_-decorated Cr_2_O_3_and Cr_2_O_3_, it was found that Mn_3_O_4_-decorated Cr_2_O_3_ MONPs had higher sensitivity compared to the other nanomaterials in Table 2 and had a low operating temperature than ZnO-Cr_2_O_3_ and SnO_2_-decorated Cr_2_O_3_, which reduces energy consumption. In addition, it has a fast response time of 20 s and a reasonable recovery time of 40.1 s, which enhances its efficiency in rapid gas detection and recovery after its removal. The superiority of this material is due to the cooperative effect between Mn_3_O_4_ and Cr_2_O_3_, as this interaction contributes to the creation of active sites that increase the sensitivity of the material and its interaction with H_2_S gas. This balanced performance makes Mn_3_O_4_-decorated Cr_2_O_3_ a suitable choice for H_2_S sensing applications with high effectiveness and efficiency.

## 5. Gas Sensing Mechanism

**Figure 7:**
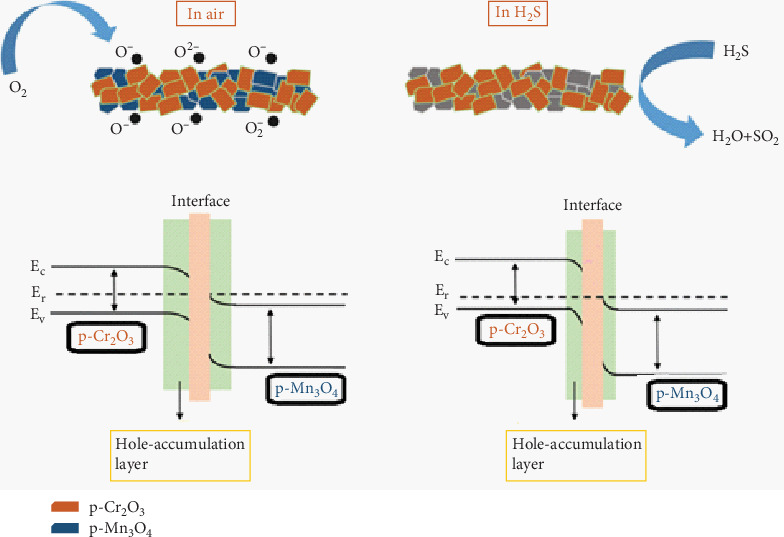
Gas sensing mechanism for Mn_3_O_4_-decorated Cr_2_O_3_ MONPs in air and H_2_S [35].

The H_2_S gas sensing process on the surface of Mn_3_O_4_-decorated Cr_2_O_3_ MONPs at a temperature of 200°C occurs through a complex mechanism involving surface adsorption and chemical reactions that affect the charge carriers in the material (Figure 7). Mn_3_O_4_ as the basic semiconductor material has hole as the main charge carriers (p-type semiconductor), while Cr_2_O_3_ acts as an effective catalyst that enhances the chemical reaction with the gas and increases the number of active sites on the surface [36, 37]. When exposed to air, oxygen is adsorbed onto the surface of the sensor, forming negatively charged oxygen ions (O_2_^−^ and O^−^) due to the sensitizing temperature being 200°C; this leads to the hole-accumulation layer (HAL) from the semiconductor material, causing an increase in the hole concentration and an increase in conduction [38, 39]. Upon exposure to H_2_S gas, the gas reacts with the adsorbed oxygen ions according to the reaction [40]:

 $$\begin{array}{c} 2{\text{H}}_{2}\text{S}+3{\text{O}}_{2}\left(\text{g}\right)⟶2{\text{H}}_{2}\text{O}\left(\text{g}\right)+2{\text{SO}}_{2}\left(\text{g}\right)+3{\text{e}}^{-}. \end{array}$$

This reaction removes the oxygen ions from the surface and releases electrons, which decrease the concentration of charge carriers in Mn_3_O_4_, leading to reduced electrical conductivity of the sensor, allowing for the detection of H_2_S gas [41, 42].

## 6. Conclusion

In this study, the photolysis method which is fast, simple, and environmentally friendly was used to prepare Mn_3_O_4_-decorated Cr_2_O_3_ MONPs. FESEM images revealed a network of Mn_3_O_4_ threads, with Cr_2_O_3_ appearing as small particles on the surface of these threads. This distribution indicates that the Cr_2_O_3_ forms a layer covering the Mn_3_O_4_ threads. The combination of Mn_3_O_4_-decorated Cr_2_O_3_ MONPs oxides significantly enhanced the sensitivity of the sensor by creating multiple active sites and enabling efficient charge transfer. Experimental results for Mn_3_O_4_-decorated Cr_2_O_3_ MONPs showed that the optimal operating temperature for detecting H₂S gas was 200°C, giving a sensitivity of 89.93. This performance was significantly higher than that of the sensor made of pure chromium and MnO_x_. The good performance of Mn_3_O_4_-decorated Cr_2_O_3_ makes it a suitable choice for sensing applications with high effectiveness and efficiency.

## Figures and Tables

**Table 1:** Sensitivity, response time, and recovery time of Cr_2_O_3_, Co_3_O_4_, and Mn_3_O_4_-decorated Cr_2_O_3_ MONP membranes at the optimum sensing temperature for H_2_S gas detection.

**Material**	**Optimum ** **T** ** (°C)**	**S** ** (%)**	**Response time (s)**	**Recovery time (s)**
Mn_3_O_4_-decorated Cr_2_O_3_	200	89.93	20	40.1
Cr_2_O_3_	200	31.88	24.3	64.8
Mn_3_O_4_	200	58.18	14.4	54.9

**Table 2:** A comparison of the gas sensing properties of the present sensor with other sensors incorporating Cr_2_O_3_ and Mn_3_O_4_ MONPs in their composition toward the H_2_S gas.

**Material**	**Operating ** **T** ** (°C)**	**S** ** (%)**	**Response time (s)**	**Recovery time (s)**	**Ref.**
Mn_3_O_4_/WO_3_	90	25	—	—	[19]
ZnO–Cr_2_O_3_	350	27	13	26	[32]
SnO_2_-decorated Cr_2_O_3_	300	30	—	—	[33]
Cr_2_O_3_	133	24.4	69	63	[34]
Mn_3_O_4_-decorated Cr_2_O_3_	200	89.93	20	40.1	This work

## Data Availability

The data that support the findings of this study are available from the corresponding author upon reasonable request.
